# Same or different? Theory of mind among children with and without disabilities

**DOI:** 10.1371/journal.pone.0202553

**Published:** 2018-10-01

**Authors:** Joanna Smogorzewska, Grzegorz Szumski, Paweł Grygiel

**Affiliations:** 1 University of Warsaw, Department of Education, Warsaw, Poland; 2 Jagiellonian University, Faculty of Philosophy, Institute of Pedagogy, Cracow, Poland; Swansea University, UNITED KINGDOM

## Abstract

Assessing theory of mind (ToM) with reliable and valid measures is important, as ToM plays a significant role in children’s social and cognitive functioning. With this in mind, a thorough analysis of the Theory of Mind scale and the Faux Pas Recognition Test was conducted. Over 750 school-age (*M* age = 7.7) children with disabilities (mild intellectual disability, hearing impairment) and without disabilities took part in our study. The psychometric properties of measures in these groups of children were checked, using confirmatory item factor analysis, reliability, and validity analyses. Thanks to groups’ invariance it was possible to compare mean results of children in the groups. Both measures showed well-fitted models with acceptable goodness of fit as well as scalar and strict invariance. An IRT analysis showed significant differences in the difficulty of the tasks in all groups, but the same order of passing tasks in comparison to other studies, conducted in Western countries, has been observed. Our results showed that the tasks were the easiest for children without disabilities, and most difficult for children with mild intellectual disability. We obtained significant and positive correlations between ToM and social skills and language abilities. The findings are discussed in relation to results from other studies in the field.

## Introduction

Theory of Mind (ToM) is defined as an ability that “involves seeing oneself and others in terms of mental states—the desires, emotions, beliefs, intentions, and other inner experiences that result in and are manifested in human actions” ([1], p. 655). ToM enables one to understand other’s mental states, as well as their behaviors and the consequences of them. ToM is meaningful for cognitive abilities (e.g., [2, 3]) and important for social functioning, especially as it plays a role in peer relations and communication with people (e.g., [4, 5, 6, 7, 8]), although the relationship between ToM and social functioning is circular: ToM influences social skills and social skills influence ToM development (e.g., [6]). ToM emerges among typically developing children as well as among children with developmental disorders. However, among children with disabilities, such as those with autism spectrum disorder (ASD), specific language impairment (SLI), intellectual disability (ID) and children with hearing impairment (HI), ToM is considered to be delayed or compromised (i.e., [9, 10, 11, 12, 13, 14, 15, 16]). Problems in ToM development have negative consequences and constrict many aspects of everyday life, such as role play, imagination, and understanding emotions, jokes and joking, intentions etc. (e.g., [17, 18, 19, 20, 21, 22]).

A great number of studies show that ToM develops over the lifespan (e.g., [1, 23, 24, 25, 26]).

However, most of the research on ToM development has been conducted with children at the preschool age (e.g., [2, 3, 27, 28, 29, 30, 31]). Although recent publications in the field highlight the need for extending the research beyond this age (e.g., [23, 24, 25, 26, 32, 33, 34]), there is still insufficient knowledge of ToM development in older children and adolescents.

Studies show that ToM depends on individual differences in factors such as language skills (e.g., [3]), social skills (e.g., [6, 8]), and executive functions (e.g., [35]), as well as on family characteristics (e.g., socioeconomic status, parental mind-mindedness, and siblings) and the environment in which the child is nurtured (e.g., [2]). Cultural differences, such as individualistic (focused on development of individuals) vs. collectivistic (focused on development of society) societies [36] are also considered as factors that affect ToM development. In individualistic countries ToM develops faster than in collectivistic ones, and different aspects of ToM emerge in different orders in those cultures [13, 25, 37, 38, 39, 40, 41, 42].

### Measuring theory of mind

Analyzing the development of ToM requires using reliable scales and measures, as ToM is quite meaningful for children’s functioning (e.g., [5, 7, 8, 43]). Many different measures exist that were prepared at the very beginning of research into ToM development (e.g., [4, 18, 44]). Many others have appeared more recently (e.g., [24, 32, 34, 45]). Nevertheless, in a great majority of studies only false belief tasks were implemented. These tasks are mainly focused on making predictions, understanding beliefs or predicting actions (e.g., [1, 27, 46]). However, a result on a single task can be misleading and insufficient for understanding a child’s actual ability level. Moreover, false belief understanding is just one of multiple aspects of ToM that emerge during the developmental span. Other aspects are also important, e.g.: diverse desires—understanding that people can have different desires about the same objects; diverse beliefs—understanding that people can have different thoughts about the same object; knowledge access—understanding that people can have limited access to knowledge; hidden emotions—understanding that internal emotions of people can be different than those shown outside; and understanding irony and idioms—understanding ambiguous situations, etc. (e.g., [13, 47, 48, 49]). Further, many tools assessing ToM development show unstable reliability (e.g., [45, 50]). There are also some doubts regarding the ecological (social) validity of the measures used most often [29, 51]. These caveats highlight the need to find measures that address the limitations described above.

The Theory of Mind scale [13, 49] and Faux Pas Recognition Test [52] cover different aspects of ToM development, are quite popular and have been tested with children with and without disabilities. We decided, therefore, to use them to conduct a thorough analysis and for comparisons of ToM development in three diverse groups of children (with and without disabilities). It is important to know whether in groups of children with and without disabilities the psychometric properties of the tools are comparable, and thus whether ToM is comparably and accurately measured in diverse populations of children. It is a non-negligible issue, however, that the measures used to assess ToM development be universal, as they must be applicable to different populations.

#### Theory of mind scale

The Theory of Mind scale was introduced by Wellman and Liu [49] and elaborated by Peterson and colleagues [13]. The scale consists of five tasks that concern:

- diverse desires (DD),
- diverse beliefs (DB),
- knowledge access (KA),
- false beliefs (FB), and
- hidden emotions (HE).

Tasks on the scale are ordered from the least difficult to the most difficult. The first two tasks are rather simple; results of previous studies show that even children as young as two years of age can solve them correctly. Subsequent tasks in the scale are more difficult. The last task concerns differentiating between feeling and showing emotions; thus, to solve the problem, children need to reason on a higher cognitive level. The results of studies show that children of primary-school age still have problems with solving this last task. Thus, the scale construction avoids “the ceiling effect.” The scale has been used in studies with children without disabilities as well as children with disabilities, including ASD, intellectual disability, and HI (e.g., [13, 14, 44]). It has been shown that children with disabilities develop ToM slower than their typically developing peers. Moreover, children with ASD follow a different pattern of answers in comparison to other groups of children [13, 14].

Analyses by Wellman and Liu [49] and Peterson et al. [13, 14] showed that the scale has good psychometric properties. It has been used in studies with children with ASD and with children with HI (native and late signers) in comparison to children without disabilities [13]. This revealed not only some differences in the level of performance among children (native signers having the highest score, late signers having the lowest) and a significant increase of performance observed with time, but also group differences in the sequence of the emergence of ToM understanding. While groups of children with hearing impairments and children without disabilities follow the pattern established in the previous study (DD>DB>KA>FB>HE; [49]), children with ASD solve the hidden emotions task before the false belief task, which means a different order of development of some aspects of ToM in this group. Further studies have encompassed between-cultures comparisons. Children from Australia, Germany, Indonesia, and the US acquire ToM in the originally assumed sequence [13, 38, 39]. However, children from Chinese and Iranian cultures develop knowledge access before diverse beliefs [26, 40]. This disparity (as touched on above) has been explained in terms of differences between individualistic versus collectivistic cultures (e.g., [36]). In a study by Zhang, Shao, and Zhang [42], the sequence noticed earlier for collectivistic cultures was reaffirmed for children without disabilities. However, for Chinese children with ASD, the pattern was similar to the ASD group from Western culture.

#### Faux Pas Recognition Test

The Faux Pas Recognition Test was introduced by Simon Baron-Cohen and colleagues [52]. It consists of 20 short stories, 10 of which contain a faux pas (where one of the characters behaves in an inappropriate way that causes embarrassment to another character). The person who committed the faux pas, however, does not realize that the behavior was inappropriate.

The story is acknowledged as understood when the child correctly answers all the questions asked.

In the first study by Baron-Cohen et al. [52], it was shown that typically developing children, aged 7–11, get better at dealing with faux pas with age, and that girls do better than boys. The ability to detect a faux pas was also examined in groups of children with Asperger Syndrome and with high-functioning autism. Results indicated that these children are significantly worse at recognizing faux pas in comparison to typically developing children, even if the comparison is conducted only with boys’ results. When answers to stories without faux pas were also taken into consideration, it was shown that typically developing children and high-functioning children with ASD solve these stories similarly and do not have major problems with them. However, the stories with faux pas, in general, were much more difficult than those without faux pas. At the same time, Baron-Cohen and colleagues assumed that stories within the same group (with/without faux pas) do not differ from each other in terms of difficulty.

### The current study

The current study has four main aims. First, we conduct psychometric analyses of both measures, using confirmatory item factor analysis, reliability and validity analyses, with results obtained from other ToM measures (Children’s Social Understanding Scale, CSUS, [31]), and on measures of social functioning and language skills in peer and school environments. Other analyses have shown that there is a strong connection between the development of ToM and social and language skills (e.g., [3, 6]); children with higher social and language skills have better developed ToM. Thus, contact with peers plays an important role in the process of ToM acquisition and the level of ToM development supports social functioning (e.g., [5, 8, 53]). Results from other ToM measures, as well as results showing development of social and language skills, are good sources of information for analyses showing external validity of chosen tools.

Moreover, we analyze the psychometric properties of measures in three groups of children: children without disabilities, children with mild intellectual disability (MID), and children with HI. Although there are studies that show how ToM develops in groups of children with disabilities (e.g., [12, 13, 14, 15, 54, 55]), there are few studies showing whether ToM tools are similarly precise in measuring ToM among children with and without disabilities. As we were not able to examine groups of children with all types of disabilities (who are known to have problems with ToM development), we chose children who on average often take part in studies on ToM development, but not as often as children with ASD: children with MID and with HI. Thus, apart from the analyses described above, we conducted an invariance analysis of all three groups to check whether between-group analysis was supported.

Third, we analyze whether and how children in the three groups differ in terms of ToM development, measured with the Theory of Mind scale and the Faux Pas Recognition Test. It is worth observing whether and how children with sensory versus cognitive impairments differ from each other with respect to ToM development. There are some studies comparing development of ToM among children with ASD and with intellectual disability [15], or with ASD and HI [13, 14]. We are, however, not acquainted with studies showing whether the development of ToM in groups of children with MID and HI is similar with respect to order of acquisition and in pace of growth. Difficulties in ToM development in these groups are caused by problems with language development, but it is not known whether these problems are responsible for the same level of ToM and whether such children need similar support in this matter. This consideration is meaningful for working with these children, especially for choosing training methods to improve their functioning. Fourth, we also prepared adaptations of analyzed measures to make them suitable for use in Poland. Currently, there are no official versions of these measures in Polish.

## Method

### Participants

Seven hundred and sixty children from different regions in Poland (*N* = 316 girls and *N* = 444 boys) took part in the study. This included children without disabilities (*N* = 243), children with MID (*N* = 249), and children with severe HI (*N* = 268), who learned in special (children with disabilities), inclusive and regular (children without disability) classrooms. Children with developmental disorders had a clinical diagnosis of a disability, prepared by specialists: psychologists and audiologists. They did not have other disorders, such as ASD or physical disability. The children with MID did not have HI, and the children with HI did not have intellectual disability. The children with HI had parents without HI–none of them was “native signer”. One hundred and forty-five children used hearings aids (*N* = 145) or implants (*N* = 74). Forty-nine children were profoundly deaf. All the children were verbal; however most of the HI children used sign language as a support in communication. All participants were learning at the elementary school level. The mean age of the children was *M* = 7.7, *SD* = .93 (5.11–12.0); children without disabilities: *M* = 7.5, *SD* = .68 (6.0–9.0); children with MID: *M* = 8.2, *SD* = .95 (6.0–12.0); and children with HI: *M* = 7.5, *SD* = .99 (5.11–10.11). The study was assessed and approved by the ethical committee at the Maria Grzegorzewska University in Warsaw, Poland (approval number 60-2014/2015). Parents of all children taking part in the study gave written permission for their child’s participation, and all children agreed to participate.

### Procedure and tasks

Before beginning the study we adapted the measures for Polish. They were translated into Polish and then back-translated. The measures for children were checked by special education teachers to ensure that the content was understandable for children with developmental delays. However, it is worth noting that in the end no changes were made to words, order of information given, or sense of the terms, in comparison with the original English text. For each story from the Theory of Mind scale, as well as from the Faux Pas Recognition Test, we added attractive pictures to make the text easier to understand. It was important for children with disabilities to visualize the main point of the story. Such elements do not influence the results: a meta-analysis by Wellman, Cross, and Watson [1] showed that the way stories are presented (i.e., verbal, verbal with pictures, with real objects, with toys etc.) does not alter the findings. Before the current study we conducted a pilot study with 72 children: without disabilities, with MID, and with HI (*N* = 22, 20, and 30, respectively), age *M* = 7.2, *SD* = 1.06 (5.11–11.0). Children solved all tasks from the Theory of Mind scale, listened to all 20 stories from the Faux Pas Recognition Test, and answered questions. The pilot study role was only to check the measures, i.e., whether the tasks were understandable for children. Analysis of the ways in which children solved the tasks helped improve the Polish adaptation and translation of the measures.

In the current study, children solved the ToM tasks during an individual meeting in a separate, quiet room. The researchers are psychologists and educators, specially trained for conducting the study and possessing experience in working with children with diverse and special educational needs. The stories were presented to the children in the same order.

In the case of the Theory of Mind scale, the order was arranged by results given by its authors [13, 49]. To correctly solve the tasks from this scale, children needed to answer every question given in the task. Depending on the story, there were two to four questions (prepared by the authors of the measure). For every task done correctly, children received one point. For all five stories, children could receive 0 to 5 points. Higher results indicate better ToM development.

The analysis of the results of the pilot study showed that the stories from the Faux Pas Recognition Test vary in terms of difficulty, i.e., not only are the stories without faux pas—in most but not in all cases—easier than stories with faux pas, but there is also variation in difficulty among the stories with and without faux pas. As such, in the current study, we chose four stories (two with/two without faux pas), which, in the pilot study, were found to be difficult (over 70% incorrect answers in the pilot study); four stories (two with/two without faux pas) which were easy (over 70% correct answers); and two stories (one with/one without faux pas) with average difficulty level (~ 50% correct and incorrect answers). In total, children listened to 10 stories. The reasons for cutting down the original number of 20 stories were problems with perception and with focusing attention among children with disabilities. Solving all 20 stories turned out to be tiresome for children. The order of presenting the stories was: a story with faux pas followed by a story without faux pas, and the stories were mixed in the level of difficulty. To earn a point for a correctly solved story, children needed to answer four questions: (a) whether there was a faux pas behavior in the story, (b) who behaved like that, (c) why it was a faux pas (justification), and (d) whether the character’s faux pas was made accidently (which is a condition for a faux pas behavior). Altogether, children could receive from 0 to 10 points on this test. A higher result indicates a better ability in accurate identification of faux pas.

### Validity measures

The following scales were used to validate the measures: a self-report scale for parents on children’s theory of mind development, Children’s Social Understanding Scale (CSUS; [31]); Taxonomy of Problematic Social Situations for Children (ToPSS; [4]), a scale for teachers to assess the ability to cope with difficult social situations. The following measures completed directly by children were also used: the Children’s Self-Efficacy for Peer Interaction Scale (CSPI; [56]), assessing how the child perceives his/her own abilities to get along with other children; the Questionnaire of Student Integration (QSI; [57]), assessing the child’s integration in school and with peers; and language tasks, chosen from the Test of Abilities at the Beginning of Elementary School (Test Umiejętności na Starcie Szkolnym, TUNSS; [58]), to check the child’s linguistic abilities. All measures, if they were not in Polish or had not previously been adapted to Polish, were translated with back-translation. All statistics shown in this section were counted for data collected in the current study.

The Children’s Social Understanding Scale was introduced by Deniz Tahiroglu and colleagues [31]. The scale consists of 42 items and six subscales concerning: beliefs, knowledge, perception, desires, intentions, and emotions. The items enable assessment of a child’s functioning in a natural context; for this reason the measure seems to be much more socially valid than others [31]. The items are assessed by parents on a 4-point Likert scale (from 1—“definitely untrue of my child” to 4—“definitely true to my child”). The answer “don’t know” is also provided, but parents are asked to use it as infrequently as possible. The psychometric analysis of the scale shows an acceptable reliability (*α* = .94, test-retest reliability: *r*(29) = .88, *p* = .001). Correlations between subscales were quite high (*r*(463) = between .55 and .76, *p* = .001, *M* = .68). The scale has been constructed as a supplemental method of examining ToM, not as a main one.

The Taxonomy of Problematic Social Situations for Children [4] consists of 44 statements, assessed by teachers on a 5-point-Likert scale, ranging from 1—the situation is never problematic for the child, to 5—the situation is always difficult for the child. The higher the points the more difficulty the child has with social skills. Factor analysis led to distinguishing six factors in this scale: (a) peer group entry, (b) response to provocation, (c) response to failure, (d) response to success, (e) social expectations, and (f) teacher expectations. Reliability of the scale was 0.96, and Pearson’s r correlation test-retest was 0.79.

The Children’s Self-Efficacy for Peer Interaction Scale [56] consists of 22 statements that the child must assess depending on whether the described situation is: very hard (1), hard (2), easy (3), or very easy (4) for him/her. According to the child’s self-assessment, the more points he/she has, the better their social skills. Factor analysis led to distinguishing two main factors, conflict and nonconflict situations. Twelve statements concern conflict situations, and 10 statements concern nonconflict situations. The reliability (Pearson’s r test-retest correlation) of the scale was *r* = 0.86.

The Questionnaire of Student Integration was adapted from a Swiss questionnaire, Fragebogen zur Erfassung von Dimensionen der Integration von Schülern FDI 4–6 [57]. The measure consists of three subscales: emotional integration (concerning the student’s emotions regarding school); social integration (concerning satisfaction regarding contact with peers); and motivation toward learning (concerning self-assessment of cognitive abilities). Students assess each item with a 4-point Likert scale, from 1—no to 4—yes. The reliability of the Polish version of the scale was satisfactory (i.e., *α* = .91).

Language tasks were taken from the Test of Abilities at the Beginning of Elementary School [58], prepared by the Educational Research Institute in Poland. The test consists of 12 tasks on recognizing letters, listening, writing, and reading skills. The reliability of the measure was acceptable (i.e., *α* = .86).

### Statistical analysis

For both measures, we conducted the analyses following the same steps:

1 For the Faux Pas Recognition Test, we calculated the reliability (Cronbach’s *α*) using tetrachoric correlation matrix for binary data. Studies show that Cronbach’s *α*, based on Pearson’s correlation matrix, “can be spuriously deflated with less than five scale points” ([59], p. 21). Hence, for binary data a better solution is to estimate *α* based on tetrachoric correlation matrix, thereby taking into account the nature of the data [59],
2 We conducted the confirmatory item factor analysis (factor loadings, item discriminations, and item difficulties) for all children and for separate groups, to see whether and how the measures fit in diverse populations. For the Faux Pas Recognition Test, we conducted separate analyses for stories with and without faux pas, as well as reliability analysis,
3 We conducted a between-groups invariance analysis, which enabled comparisons between groups. A measurement invariance is a critical assumption in every longitudinal or between-groups comparison [60]. Its aim is to verify an assumption that the observed result is independent from group belongingness (gender of participants) and depends only on the level of a studied construct [61, 62]. A lack of at least partial measurement invariance makes it impossible to interpret differences among a given model’s parameters, among next waves of the study and/or between groups [63]. Researchers agree, for example, that a sense of comparison of differences between means must be based on at least partial scalar invariance [9, 64, 65], assuming that not only means of items (in the case of qualitative variables—thresholds) but also factor loadings do not differ significantly between groups [62]. However, using composite scores (summed or averaged results) in analyses requires at least partial strict invariance.

To test measurement invariance we used a procedure of sequence estimation of series hierarchical nested models with a growing number of constraints. The first tested model was a configural model (M0), a model in which there is no assumption about invariance of a measurement tool among three analyzed groups (all parameters are tested as potentially independent from each other). At this level we were only interested in whether the same items measured the same construct across groups.

In the second step we estimated a metric model (M1) with constraint on between-groups’ factor loadings (i.e., weak invariance). In the third step we estimated a scalar model (M2), in which—apart from factor loadings—we also constrained thresholds of items. If a factor model is presented as a regression equation [66], factor loadings are responsible for the slope of regression line of observed to latent result, and means of items (thresholds) show whether a center of latent variable in different groups is in the same place. As a consequence, a lack of equality of factors and/or means (thresholds) indicates a lack of a parallel between regression lines, and a need to reject a hypothesis that in analyzed groups the same observed result is connected with the same level of latent variable.

The next tested type of invariance was a strict invariance, which additionally assumes an approximate equality of residual errors in tested items. In this model it is assumed that differences in observed variables’ variance result only from differences in latent variables variance, and errors’ variance do not differ in analyzed groups. The strict invariance, therefore, is an indicator of between-groups reliability of measurement and implies at the same time that “group differences in manifest variable means and variances are accounted for by group differences in common factor means and dispersion matrices” ([67], p. S72).

4 Finally, we checked the validity of the measures using Pearson’s r correlation matrix.
  Analyses were prepared with Mplus 7.4 [68] and IBM SPSS 24. In the analysis a Weighted Least Squares Means and Variance Adjusted (WLSMV) estimator, based on tetrachoric correlation matrix, was used. WLSMV is better than other estimators (such as maximum likelihood), for analyzing dichotomous data in small- to medium-size samples.

## Results

### Observed item difficulties

In the preliminary analysis, we checked how many children passed ToM tasks, globally and in each group. The percentage of children who passed each of the tasks is presented in Table 1.

**Table 1:** A percentage of children passing theory of mind tasks.

Task	All children	Children without disabilities	Children with MID	Children with HI
Diverse Desires Diverse Beliefs Knowledge Access False Belief Hidden Emotions Faux Pas 1* Faux Pas 2 Faux Pas 3* Faux Pas 4 Faux Pas 5* Faux Pas 6 Faux Pas 7* Faux Pas 8 Faux Pas 9* Faux Pas 10	76.3 72.3 63.2 44.4 31.8 35 38.8 24 39.7 48.6 29 18.3 25.9 41.5 53.6	83.6^a^ 82.8^a^^b^ 80.8^a^^b^ 64.4^a^^b^ 52^a^^b^ 56.4^a^^b^ 38.8 34.4^a^^b^ 51.6^a^^b^ 72^a^^b^ 34^a^ 25.6^a^^b^ 25.6 66^a^^b^ 58^a^	69^a^ 65.5^a^ 53.1^a^ 26^a^^c^ 15.1^a^^c^ 13.6^a^^c^ 36.4 15.1^a^ 29.8^a^ 27.9^a^^c^ 22.1^a^ 12.8^a^ 26.7 19.4^a^^c^ 43^a^^c^	76.5 69.1^b^ 56.6^b^ 43.4^b^^c^ 29^b^^c^ 35.7^b^^c^ 41.2 22.8^b^ 38.2^b^ 46.7^b^^c^ 30.9 16.9^b^ 25.4 40.1^b^^c^ 59.6^c^

Note: Tasks with asterix (*) indicate stories with faux pas. MID–mild intellectual disability, HI–hearing impairment

^a^ significant differences between children without disabilities and children with MID, *p* < 0.05

^b^ significant differences between children without disabilities and children with HI, *p* < 0.05

^c^ significant differences between children with MID and with HI, *p* < 0.05.

Although the analysis is simple and based on percentages, a few interesting points emerged. First, it can be clearly observed that the difficulty of tasks in the Theory of Mind scale, in all three groups, followed the same pattern as in the study by Wellman and Liu [49] and other studies conducted within Western societies. However, final conclusions can be made after analyzing results of Item Response Theory (IRT). Overall, children without disabilities passed the Theory of Mind scale tasks more often than children with disabilities, and children with MID were the weakest. In the case of the Faux Pas Recognition Test, children without disabilities again outperformed children with disabilities, with children with MID achieving the lowest result. However, the differences in passed versus non-passed tasks between groups varied from very small and insignificant (Faux Pas 2 & 8) to moderate and significant (Faux Pas 1*, 5*, 9*).

### Main analysis

#### Theory of mind scale

**Factor analysis**

The analysis of goodness of fit for a one-factor basic model showed some room for improvement. The *RMSEA* for a general model (for all children) was .08 (*CFI* = .953, *TLI* = .907). However, the indices of goodness of fit were less acceptable in the group of children without disabilities (*RMSEA* = .087, *CFI* = .892, *TLI* = .784) and in the group of children with MID (*RMSEA* = .076, *CFI* = .900, *TLI* = .800). Only in the group of children with HI was the fit of the model very good (*RMSEA* = .03, *CFI* = .994, *TLI* = .989), showing that the result in this group was responsible for an overall acceptable effect. That is why we improved the model using values of modification indices. The most reasonable suggestion was to correlate the first task with the second one (M.I. for general model = 28.100, for children without disabilities = 10.600, for children with MID = 10.433, for children with HI = 3.366). This change seemed to make sense, because these tasks are similar and are the simplest tasks in the scale. The change brought a visible positive change in the model fit. The goodness of fit indices and estimates for all the models and items are presented in Table 2. *Chi*^*2*^ has a value higher than 0.05, RMSEA is less than 0.05, and CFI and TLI are higher than 0.95, for all children as well as for each group.

**Table 2:** The ToM scale. A modified model (ToM1 correlated with ToM2).

	All children (*N* = 760)			Children WD (*N* = 243)			Children with MID (*N* = 249)			Children with HI (*N* = 268)		
	Factor loadings -STDYX	Item Discriminations	Item Difficulties	Factor loadings STDYX	Item Discriminations	Item Difficulties	Factor loadings—STDYX	Item Discriminations	Item Difficulties	Factor loadings—STDYX	Item Discriminations	Item Difficulties
ToM1	.48(.06)	.55(.09)	-1.50(.22)	.40(.14)	.43(.18)	-2.47(.92)	.38(.13)	.41(.16)	-1.33(.50)	.58(.09)	.72(.16)	-1.28(.25)
ToM2	.54(.05)	.64(.09)	-1.10(.15)	.42(.13)	.46(.18)	-2.23(.77)	.35(.11)	.37(.14)	-1.17(.45)	.67(.08)	.90(.19)	-.76(.15)
ToM3	.75(.05)	1.12(.17)	-.47(.07)	.67(.15)	.90(.37)	-1.31(.33)	.68(.13)	.93(.33)	-.13(.12)^NS^	.77(.07)	1.19(.26)	-.23(.10)
ToM4	.75(.05)	1.13(.17)	.18(.06)	.67(.14)	.90(.34)	-.53(.16)	.65(.13)	.85(.30)	.97(.24)	.78(.07)	1.24(.29)	.19(.10)
ToM5	.52(.06)	.61(.09)	.90(.14)	.24(.13)^1^	.25(.14)^1^	-.28(.37)^NS^	.47(.14)	.53(.20)	2.22(.20)	.50(.09)	.58(.14)	1.09(.69)
Goodness of fit:												
χ^2^ (df)	1.892(4); p = .755			3.523(4); p = .474			1.977(4); p = .739			3.084(4); p = .544		
RMSEA	.000 [.000-.038]			.000 [.000-.092]			.000 [.000-.068]			.000 [.000-.082]		
CFI	1.000			1.000			1.000			1.000		
TLI	1.000			1.000			1.000			1.000		

Note: All values significant at p < .05, except these with ^1^ –p < .1 and ^NS^–non significant. Children WD–children without disabilities, Children with MID–Children with mild intellectual disability, Children with HI–children with hearing impairment.

**IRT parameters**

An analysis of IRT parameters showed that the first three tasks were easy for all children (the first task—“diverse desires” being the easiest—Item difficulty is the location of item on latent trait level at which one has a 50% probability of response = 1. Negative values indicate, though, a modest difficulty of a given item). The fourth (“false belief”) and fifth (“hidden emotion”) tasks were still rather easy for children without disabilities; the fifth task was the most difficult. For children with MID, the fourth and fifth tasks were much more difficult than the first three tasks. There was a similar result in the group of children with HI, but differences in difficulty between the first three tasks and last two tasks were smaller. The scale composition and the order of task difficulty in the scale were consistent with previous results. It was also comparable for all three subsamples within the current study (Table 2).

**Invariance analysis**

More importantly, after conducting between-groups analysis of invariance, we were able to obtain both a scalar invariance (equality of factor loadings and thresholds) and a strict invariance (equality of factor loadings, thresholds, and residual variances) after modification. To obtain a strict invariance we needed to release residual variances for the “hidden emotion” task (ToM 5) in the group of children with MID (M.I. = 10.975). The model with constraints was not significantly worse than the model without constraints. The results of the analysis are presented in Table 3. They enable between-groups mean comparisons.

**Table 3:** Model fit indices for the multigroup measurement invariance models of the ToM scale.

*Model*	*χ*^*2*^ *(df)*	*p*	*Δ χ*^*2*^ *(df)*	*p*	*RMSEA*	*RMSEA 90% CI*	*CFI*	*TLI*
Configural^a^	8.667(12)	0.731	-	-	0.000	0.000–0.047	1.000	1.000
Scalar^b^	13.379(18)	0.768	4.535(6)	0.604	0.000	0.000–0.039	1.000	1.000
Strict^c^	37.416(28)	0.11	21.776(10)	0.016	0.036	0.000–0.064	0.977	0.976
Strict^d^	26.711(27)	0.479	12.336(9)	0.195	0.000	0.000–0.048	1.000	1.000

Note. χ2 = chi-square goodness-of-fit statistic; *df* = degrees of freedom; TLI = Tucker–Lewis Index; CFI = comparative fit index; RMSEA = root mean square error of approximation; CI = confidence interval

^a^ Factor loadings and thresholds freely estimated

^b^ Factor loadings and thresholds constrained

^c^ Factor loadings, thresholds and residual variances constrained (before modification)

^d^ Factor loadings, thresholds and residual variances constrained (after modification).

**Fig 1:**
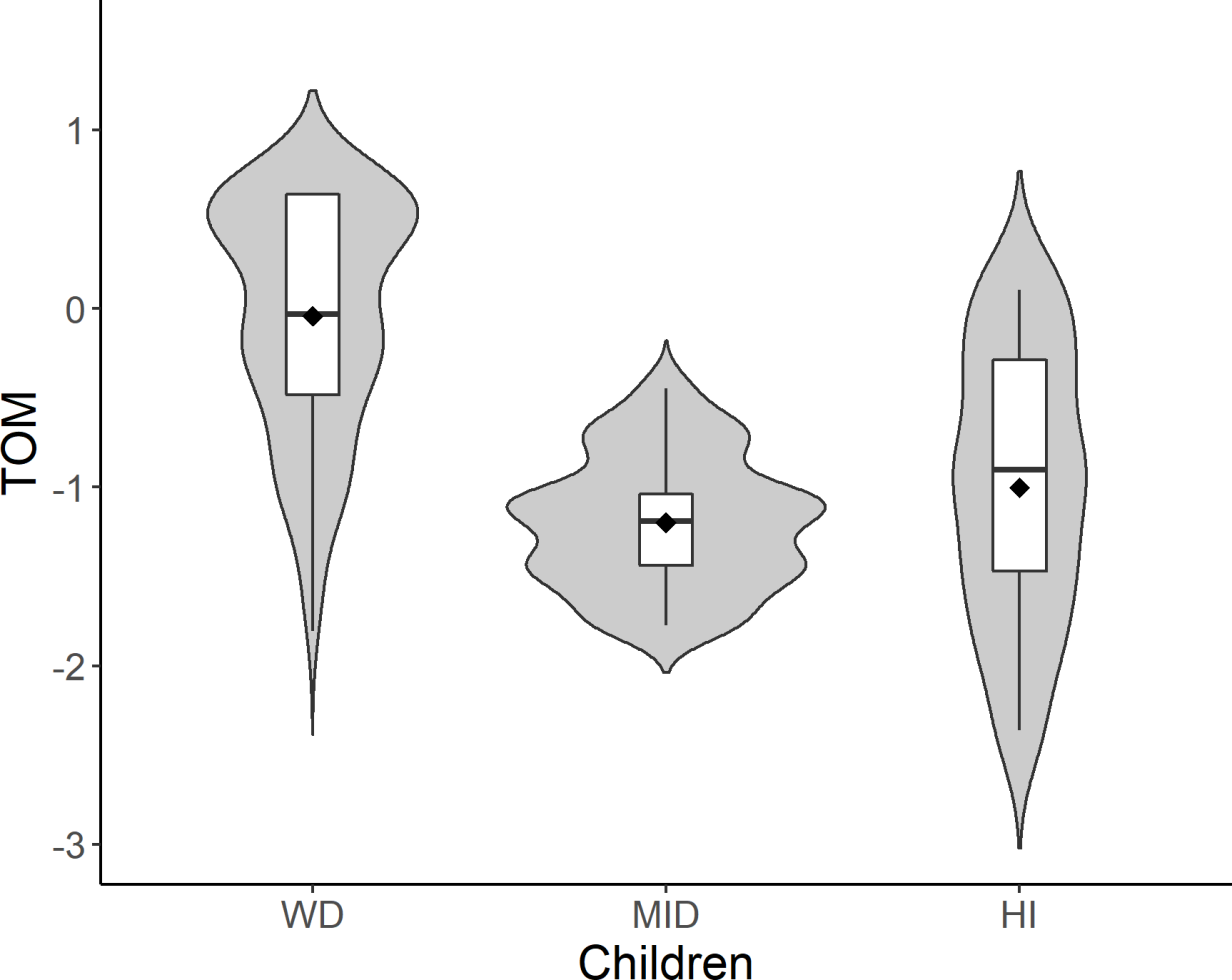
The ToM scale results. Note. Children WD–children without disabilities, Children MID–children with mild intellectual disabilities, Children with HI–children with hearing impairment.

The analysis showed that, in comparison to children without disabilities, children with MID (*M* = -1.209, *SE* = .192) and with HI (*M* = -.991, *SE* = .172) achieved significantly lower results (*p* < .01). The results are presented in Fig 1.

#### Faux pas recognition test

The analysis was prepared separately for stories with and without faux pas.

**Reliability**

The analysis of reliability of faux pas stories brought moderately satisfactory results, which can be caused by the length of both subscales. In the case of the first subscale (stories with faux pas) for all children it was *α* = 0.83, for children without disabilities *α* = 0.75, for children with MID *α* = 0.78, and for children with HI*α* = 0.82. In the case of the second subscales (stories without faux pas) for all children it was *α* = 0.70, for children without disabilities *α* = 0.67, for children with MID *α* = 0.69, and for children with HI *α* = 0.72.

**Factor analysis**

The goodness of fit for a general model of stories with faux pas was good. It was a bit weaker in the group of children with HI. The goodness of fit for a general model for stories without *faux pas* was even better, however, and again it was weakest in the group of children with HI. The goodness of fit indices and estimates are presented in Table 4 and Table 5.

**Table 4:** The Faux Pas Recognition Test. The subscale with stories with *faux pas*.

	All children (*N* = 760)			Children WD (*N* = 243)			Children with MID (*N* = 249)			Children with HI (*N* = 268)		
	Factor loadings—STDYX	Item Discriminations	Item Difficulties	Factor loadings—STDYX	Item Discriminations	Item Difficulties	Factor loadings—STDYX	Item Discriminations	Item Difficulties	Factor loadings—STDYX	Item Discriminations	Item Difficulties
FP1	.77 (.04)	1.22 (.14)	.48 (.07)	.74 (.09)	1.10 (.30)	-.26 (.11)	.70 (.09)	.97 (.23)	1.57 (.25)	.72 (.07)	1.03 (.20)	.51 (.12)
FP3	.64 (.05)	.84 (.09)	1.07 (.11)	.59 (.08)	.73 (.15)	.64 (.18)	.64 (.09)	.83 (.20)	1.58 (.28)	.67 (.07)	.89 (.18)	1.10 (.18)
FP5	.81 (.03)	1.41 (.19)	.03 (.06)^NS^	.58 (.10)	.71 (.19)	-1.03 (.23)	.89 (.07)	1.97 (.70)	.62 (.11)	.81 (.06)	1.36 (.31)	.12 (.10)^NS^
FP7	.49 (.05)	.56 (.08)	1.84 (.23)	.53 (.10)	.62 (.16)	1.26 (.30)	.32 (.11)	.34 (.13)	3.49 (1.24)	.55 (.08)	.66 (.14)	1.73 (.32)
FP9	.82 (.04)	1.44 (.19)	.24 (.06)	.64 (.10)	.83 (.22)	-.69 (.16)	.79 (.07)	1.27 (.31)	1.09 (.16)	.79 (.07)	1.29 (.29)	.31 (.10)
Goodness of fit:												
χ^2^ (df)	8.306 (5); p = .14			2.661 (5); p = .75			8.439 (5); p = .13			12.170 (5); p = .03		
RMSEA	.029 [C.I. = .000-.064]			.000 [C.I. = .000-.062]			.053 [C.I. = .000-.112]			.073 [C.I. = .020-.127]		
CFI	.997			1.000			.984			.979		
TLI	.994			1.000			.969			.957		

Note: All values significant at p < .05, except these with ^1^ –p < .1 and ^NS^–non significant. Children WD–children without disabilities; Children with

MID–Children with mild intellectual disability; Children with HI–children with hearing impairment.

**Table 5:** The Faux Pas Recognition Test. The subscale with stories without *faux pas*.

	All children (*N* = 760)			Children WD (*N* = 243)			Children with MID (*N* = 249)			Children with HI (*N* = 268)		
	Factor loadings—STDYX	Item Discriminations	Item Difficulties	Factor loadings—STDYX	Item Discriminations	Item Difficulties	Factor loadings—STDYX	Item Discriminations	Item Difficulties	Factor loadings—STDYX	Item Discriminations	Item Difficulties
FP2	.65 (.05)	.86 (.12)	.43 (.08)	.47 (.10)	.54 (.15)	.59 (.22)	.65 (.09)	.85 (.21)	.55 (.15)	.86 (.07)	1.68 (.56)	.25 (.09)
FP4	.61 (.05)	.77 (.11)	.45 (.09)	.56 (.10)	.67 (.17)	-.07 (.15)^NS^	.56 (.10)	.68 (.17)	.99 (.24)	.65 (.08)	.84 (.17)	.49 (.14)
FP6	.68 (.05)	.92 (.13)	.80 (.10)	.72 (.10)	1.04 (.31)	.54 (.14)	.75 (.09)	1.12 (.29)	1.03 (.18)	.59 (.08)	.74 (.16)	.82 (.18)
FP8	.31 (.06)	.33 (.07)	2.11 (.45)	.50 (.11)	.58 (.17)	1.31 (.34)	.27 (.11)	.28 (.12)	2.46 (1.08)	.25 (.09)	.26 (.10)	2.64 (1.06)
FP10	.59 (.06)	.72 (.11)	-.18 (.08)	.48 (.11)	.54 (.16)	-.45 (.20)	.55 (.10)	.66 (.18)	.30 (.16)^1^	.63 (.08)	.82 (.18)	-.04 (.13)
Goodness of fit:												
χ^2^ (df)	3.912 (5); p = .56			4.730 (5); p = .45			2.070 (5); p = .84			10.644 (5); p = .06		
RMSEA	.000 [C.I. = .000-.044]			.000 [C.I. = .000-.087]			.000 [C.I. = .000-.050]			.065 [C.I. = .000-.119]		
CFI	1.000			1.000			1.000			.970		
TLI	1.000			1.000			1.000			.940		

Note: All values significant at p < .05, except these with ^1^ –p < .1 and ^NS^–non significant. Children WD–children without disabilities; Children with

MID–children with mild intellectual disability; Children with HI–children with hearing impairment.

**IRT parameters**

An analysis of IRT parameters showed that, as in the case of the preliminary analysis, there were some differences in the difficulty of the stories within groups. The results showed that, in general, the stories were not very difficult for the group of children without disabilities, except one story with faux pas (FP7) and one without faux pas (FP8). For children with MID the stories were visibly difficult. In particular, stories FP5 and FP7 (with faux pas) and FP8 and FP10 (without faux pas) seemed to be very challenging for them. For children with HI, the FP7 and FP8 stories turned out to be especially difficult.

**Invariance analysis**

For both subscales of stories, we were able to obtain a scalar invariance (equality of factor loadings and thresholds) and a strict invariance (equality of factor loadings, thresholds, and residual variances). The subscale with faux pas did not need any improvements, and, in the case of the subscale without faux pas, we needed to release the first item (FP2) to achieve the scalar invariance (M.I. = 11.484). After doing so, in the case of both models, configural models were not significantly worse than scalar and strict models. The results of the analysis are presented in Table 6. They enable comparison of the mean results between groups.

**Table 6:** Model fit indices for the multigroup measurement invariance models of the Faux Pas Recognition Test.

*Model*	*χ*^*2*^ *(df)*	*p*	*RMSEA*	*Δ χ*^*2*^ *(df)*	*p*	*RMSEA 90% CI*	*CFI*	*TLI*
			Subscale 1 –stories with faux pas					
Configural^a^	23.516(15)	0.073	0.047	-	-	0.000–0.082	0.988	0.975
Scalar^b^	28.633(21)	0.123	0.038	6.177(6)	0.403	0.000–0.070	0.989	0.984
Strict^d^	46.41(31)	0.037	0.044	17.896(10)	0.056	0.011–0.069	0.978	0.978
			Subscale 2 –stories without faux pas					
Configural^a^	17.272(15)	0.303	0.024	-	-	0.000–0.067	0.994	0.988
Scalar^b^	32.784(21)	0.048	0.047	14.284(6)	0.026	0.004–0.077	0.968	0.954
Scalar^c^	20.310(19)	0.376	0.016	3.393(4)	0.494	0.000–0.058	0.996	0.994
Strict^d^	36.522(27)	0.104	0.037	4.952(6)	0.550	0.000–0.066	0.974	0.971

Note. χ2 = chi-square goodness-of-fit statistic; *df* = degrees of freedom; TLI = Tucker–Lewis Index; CFI = comparative fit index; RMSEA = root mean square error of approximation; CI = confidence interval

^a^ Factor loadings and thresholds freely estimated

^b^ Factor loadings and thresholds constrained (without modifications).

^c^ Factor loadings and thresholds constrained (after modification)

^d^ Factor loadings, thresholds and residual variances constrained.

**Fig 2:**
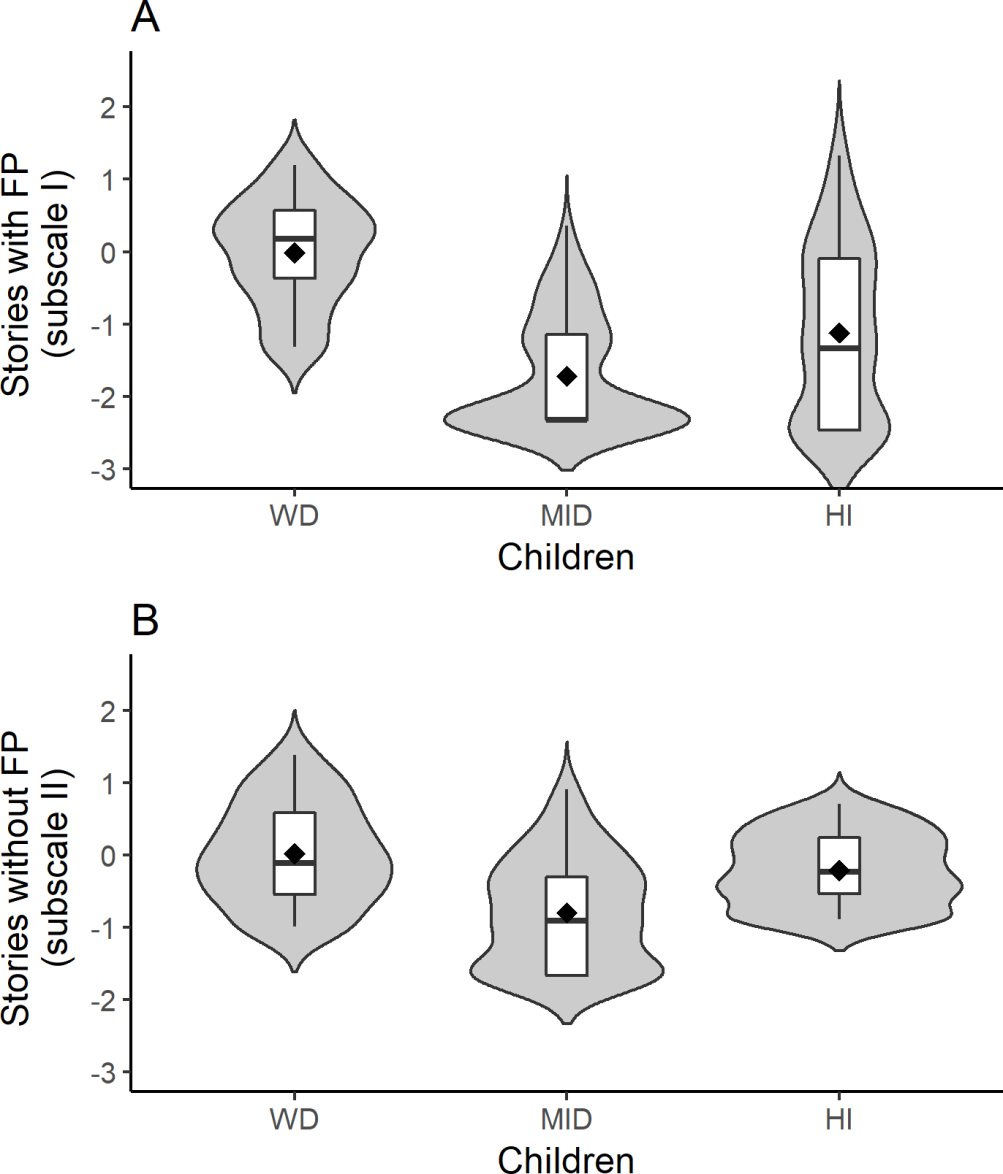
The Faux Pas Recognition Test results. A–stories with faux pas. B–stories without faux pas. Note. Children WD–children without disabilities, Children MID–children with mild intellectual disabilities, Children with HI–children with hearing impairment.

In the case of results from the subscale of stories with faux pas, the analysis showed that, in comparison to children without disabilities, children with MID (*M* = -1.846, *SE* = .380) and with HI (*M* = -1.176, *SE* = .240) achieved significantly lower results (*p* < 0.01). Results from the subscale with stories without faux pas showed that, in comparison to children without disabilities, children with MID did significantly worse (*M* = -.830, *SE* = .347, *p* < 0.01). However, children with HI attained results similar to children without disabilities (*M* = -.222, *SE* = .154, *p* = .15). These are illustrated in Fig 2.

### External validity

The last step in our analysis was to correlate ToM measures with the other measure of ToM (CSUS), as well as with measures of social (CSPI, QSI, ToPSS) and language skills. We prepared a correlation matrix with Bonferroni adjustment to control for Type I Error. The Bonferroni adjustment was counted with”corr.test” function in “psych” package, working in the R environment. The analysis revealed significant, moderate, and positive relations between ToM measures and other measures used for this analysis (Table 7).

**Table 7:** Means, standard deviations, and correlations for all children.

Variable	*M*	*SD*	1	2	3	4	5	6	7	8
1. ToM	0.58	0.29								
2. FP	0.35	0.21	.39**							
3. FPa	0.33	0.31	.42**	.75**						
4. FPb	0.37	0.28	.13**	.70**	.05					
5. CSUS	2.86	0.58	.39**	.36**	.37**	.15**				
6. CSPI	3.32	0.64	.26**	.27**	.22**	.16**	.20**			
7. QSI	3.05	0.50	.11	.19**	.14**	.13**	.20**	.21**		
8. LANGUAGE	0.74	0.27	.41**	.40**	.39**	.19**	.43**	.25**	.22**	
9. ToPSS	3.61	0.73	.20**	.20**	.22**	.07	.21**	.17**	.23**	.23**

*Note*. ToM–theory of mind scale, FP–faux pas recognition test, FPa–faux pas stories with faux pas, FPb–faux pas stories without faux pas, CSUS–Children Social Understanding Scale, CSPI–Children’s Self-Efficacy for Peer Interaction Scale, QSI—Questionnaire of Student Integration, LANGUAGE–language skills, ToPSS–Taxonomy of Problematic Social Situations

* indicates *p* < .05

** indicates *p* < .01. *M* and *SD* are used to represent mean and standard deviation, respectively.

ToM measures correlated with each other significantly, and, as expected, correlations between stories without faux pas and ToM measures were the weakest. However, moderate correlations indicate that the tested measures assessed different aspects of ToM. The Theory of Mind scale correlated more strongly with the language measure than with social skills measures. However, again, this pattern could be expected because of the significant verbal component in ToM tasks. There was no correlation between the Theory of Mind scale and the QSI. The correlations between Faux Pas Recognition Test and other measures were very similar to those observed for the Theory of Mind scale. The correlation between the QSI and Faux Pas Recognition Test is the lowest; however, it was significant.

## Discussion

In the current study we conducted a psychometric analysis of two ToM measures—the Theory of Mind Scale and the Faux Pas Recognition Test. We were able to observe whether Polish children, with and without disabilities, follow the pattern of the Theory of Mind scale first shown by Wellman and Liu [49]. We also compared three groups of children—those without disabilities, those with MID, and those with HI—in terms of the level of ToM performance.

The analysis revealed that, for both measures, it is possible to show well-fitted models. Initial results left some room for improvement in the Theory of Mind scale; however, a small change, adding correlation between the first and second items of the scale, let us achieve satisfactory goodness of fit in all three groups. In the case of the Faux Pas Recognition Test, the models did not need any improvement; they were acceptable without any modifications. However, in the group of children with HI, the goodness of fit was the lowest, though still acceptable.

A very simple analysis of percentage of passed tasks, as well as much more robust IRT analysis, showed significant differences in the difficulty of the tasks. We have observed the same order of passing tasks as shown in previous analyses conducted in other countries, such as Australia, Germany, Indonesia, and the US [13, 38, 39]. Although Poland represents a specific tension between being an individualistic society and having a considerably high level of power distance (according to which individuals in a society accept that power is not distributed equally) [36], in the case of ToM development, it seems that it follows the pattern of individualistic, Western countries. However to confirm this result, it requires further analyses.

The percentage of passed tasks shows that some of the faux pas stories not only differ from each other in their degree of difficulty; some of them are easier to pass than the tasks on the Theory of Mind scale. The result is at least different from Baron-Cohen and colleagues’ analysis [52], which showed that stories without faux pas are not always easier than those with faux pas. Rather, the results are very diverse: Some of the stories are much more difficult than others. Moreover, while Baron-Cohen and colleagues stated that most of the typically developing children, from 9 to 11, were able to pass all the control stories (without faux pas), this was not the case for the Polish children. The reasons for this may be similar to those in the case of the Theory of Mind scale. However, it is striking that some of the faux pas stories are easier to pass than those from the Theory of Mind scale. This is probably not because the recognition of faux pas is always more difficult than tasks on the scale, but, instead, because they demand paying attention to other details and aspects of the situation. As has been shown, the measures are moderately correlated, but they do not have the tendency to overlap, thus indicating some clear differences.

In our study we were able to reach scalar and strict invariance for both measures, meaning that we could compare groups in the case of their Theory of Mind results. The analysis for both measures revealed that children without disabilities achieved highest results regardless of the measure. Children with MID displayed the lowest level of ToM development for both measures. Children with HI stood in between the remaining groups. The differences between children with and without disabilities were highly expected, as they have been reported in numerous previous analyses [12, 14, 15, 41, 69]. However, we were not certain about differences and their directions in the case of children with MID in comparison with HI, as groups of this kind had not been directly compared before. It is probable that the cognitive problems observed in children with MID are more restrictive in the case of taking perspective of others than the sensory problems experienced by children with HI experience, even though the relationship between ToM and intelligence is, at least, ambiguous (e.g., [70, 71]).

The significant and positive correlations between ToM and social skills and language abilities support the well-established results from other studies (e.g., [3, 6, 39]) about the role of these capabilities in ToM development. The moderately high, but not overlapping correlations between ToM measures show the validity and usefulness for assessing ToM.

### Limitations and conclusion

Our study has a number of potential strengths. One is the relatively large sample of over 750 children. We showed the psychometric properties of two measures to assess ToM development using robust methods of analysis. Further, we were able to compare three different groups of children regarding ToM development within the Polish population of elementary school children.

Nevertheless, some limitations of the study should be mentioned. First, despite the well-fitted models obtained in our analysis, the reliability of the Faux Pas Recognition Test is not sufficiently strong to accept it without any doubts. Obviously, it is not critically low—for the entire group the measure reached the level of 0.70—however, there is still a need for improvement. The discussion about the reliability of the measures used for ToM assessment is a critical point in many papers. This is especially true when considering that results are not stable between studies and measures (e.g., [24, 45]). Moreover, the measures used in our study for validity analysis are not the classic ones. For example, the Taxonomy of Social Problematic Situations for Children does not assess concrete social skills, but, rather, a tendency of the child to show unsuitable and socially disapproved behaviors. Other measures of social skills are self-report scales, which can raise doubts about the validity of the assessment. Taking these problems into account, we decided to use a few different measures of social skills and the sense of being integrated with peers, in line with the suggestions about the multi-method assessment of social skills (e.g., [72]). However, these measures turned out to be significantly—though not very strongly—correlated, showing at least some links between each other and between them and ToM. Linguistic abilities were also not measured in a standard way. However, the children seemed to be too old to use a test of receptive language prepared for preschool children. Furthermore, this test is not appropriate for some children with HI [14]. Since all the children were at the same school education level, we decided to use a test of language knowledge that was appropriate for all children and also was easy to conduct with such a sample. In analyzing the results, it was also necessary to take into consideration the fact that children with special educational needs, even those with the same diagnosis or those seen as having a similar level of social and cognitive functioning, are diverse in the causes of their problems and delays. That is why individual results of each child can vary greatly from those of others.

Even taking into account the above caveats, the results are important not only for theory but also for educational and psychological practices, showing the value of the assessed measures not only in conducting studies with populations without disability, but also, and even primarily, with children with special educational needs.
